# Deciding How to Stay Independent at Home in Later Years: Development and Acceptability Testing of an Informative Web-Based Module

**DOI:** 10.2196/humanfactors.8387

**Published:** 2017-12-14

**Authors:** Mirjam Marjolein Garvelink, C Allyson Jones, Patrick M Archambault, Noémie Roy, Louisa Blair, France Légaré

**Affiliations:** ^1^ Centre de recherche sur les soins et les services de première ligne de l'Université Laval Centre intégré universitaire de santé et services sociaux de la Capitale-Nationale Québec City, QC Canada; ^2^ Department of Physical Therapy Faculty of Rehabilitation Medicine University of Alberta Edmonton, AB Canada; ^3^ Centre intégré de santé et de services sociaux de Chaudière-Appalaches Centre hospitalier affilié universitaire Hôtel-Dieu de Lévis Lévis, QC Canada; ^4^ Population Health and Optimal Health Practice Research Unit Centre hospitalier universitaire de Québec Université Laval Québec City, QC Canada; ^5^ Division of Critical Care Medicine Department of Anesthesiology and Critical Care Medicine Université Laval Québec City, QC Canada; ^6^ School of Architecture Faculty of Planning, Architecture, Arts and Design Université Laval Québec City, QC Canada; ^7^ Department of Family Medicine and Emergency Medicine Faculty of Medicine Université Laval Québec City, QC Canada

**Keywords:** decision making, shared, housing for the elderly, decision support techniques, instruction films and videos

## Abstract

**Background:**

Seniors with loss of autonomy may face decisions about whether they should stay at home or move elsewhere. Most seniors would prefer to stay home and be independent for as long as possible, but most are unaware of options that would make this possible.

**Objective:**

The study aimed to develop and test the acceptability of an interactive website for seniors, their caregivers, and health professionals with short interlinked videos presenting information about options for staying independent at home.

**Methods:**

The approach for design and data collection varied, involving a multipronged, user-centered design of the development process, qualitative interviews, and end-user feedback to determine content (ie, needs assessment) in phase I; module development (in English and French) in phase II; and survey to test usability and acceptability with end users in phase III. Phase I participants were a convenience sample of end users, that is, seniors, caregivers, and professionals with expertise in modifiable factors (eg, day centers, home redesign, equipment, community activities, and finances), enabling seniors to stay independent at home for longer in Quebec and Alberta, Canada. Phase II participants were bilingual actors; phase III participants included phase I participants and new participants recruited through snowballing. Qualitative interviews were thematically analyzed in phase II to determine relevant topics for the video-scripts, which were user-checked by interview participants. In phase III, the results of a usability questionnaire were analyzed using descriptive statistics.

**Results:**

In phase I, interviews with 29 stakeholders, including 4 seniors, 3 caregivers, and 22 professionals, showed a need for a one-stop information resource about options for staying independent at home. They raised issues relating to 6 categories: cognitive autonomy, psychological or mental well-being, functional autonomy, social autonomy, financial autonomy, and people involved. A script was developed and evaluated by participants. In phase II, after 4 days in a studio with 15 bilingual actors, 30 videos were made of various experts (eg, family doctor, home care nurse, and social worker) presenting options and guidance for the decision-making process. These were integrated into an interactive website, which included a comments tool for visitors to add information. In phase III (n=21), 8 seniors (7 women, mean age 75 years), 7 caregivers, and 6 professionals evaluated the acceptability of the module and suggested improvements. Clarity of the videos scored 3.6 out of 4, length was considered right by 17 (separate videos) and 13 participants (all videos together), and 18 participants considered the module acceptable. They suggested that information should be tailored more, and that seniors may need someone to help navigate it.

**Conclusions:**

Our interactive website with interlinked videos presenting information about options for staying independent at home was deemed acceptable and potentially helpful by a diverse group of stakeholders.

## Introduction

Most seniors want to live at home and remain independent for as long as possible, a goal reflected in many government policies [1,2]. Independent living encompasses a holistic concept of autonomy that includes the social, psychological, functional, and health care needs of seniors related to active aging [3]. In 2011, 92% of all Canadian seniors aged 65 years and older lived autonomously in private households [4], of whom the majority were house owners [5]. Most still drove their cars, which was their main means of transportation; fewer than 6% used public transport; and fewer than 3% walked or cycled [4]. Similar patterns are seen abroad [6,7]. However, because of age-related decline in health and autonomy, many seniors receive informal or formal care at home (30.1% aged 75-84 years, and 54% over 85 years) [8]. Moreover, most seniors and their caregivers will ultimately face a decision about whether they can continue living at home, and if so, how to maintain their independence [8,9].

Although there are multiple options for seniors to remain at home [10-12], many seniors and their caregivers are unaware of them [13,14]. Before deciding to move elsewhere, it is important that seniors know their options about aging safely in situ and weigh these options alongside the option to relocate [15]. Moreover, active involvement in decision making is key to helping people self-manage their health.

According to the Ottawa Decision Support Framework, people cannot make preference-based shared decisions without accurate knowledge of the options, an understanding of what is most important to them, and effective support from others [15]. In the decision to stay at home or relocate, different kinds of knowledge (eg, medical, social, financial, and familial) are needed and supplied by different stakeholders. A senior may prefer to stay at home, for example, whereas others may know it is unsafe or untenable for their caregiver. Although the final decision is preferably the senior’s, it is important that all the stakeholders [16-18] are involved in informing and discussing the decision. A recent ongoing study on the implementation of a shared decision-making (SDM) guide for seniors and caregivers about whether to stay at home or move indicates that the SDM guide helps stakeholders be more involved in the decision [18]. However, the guide does not provide detailed information on seniors’ options for remaining at home [19]. In addition, it is paper-based and so presentation of options is limited. Some European countries have developed Web-based tools for seniors that offer more flexibility [20,21]. We therefore developed a Web-based interactive decision support module for seniors, caregivers, and health professionals in 2 Canadian provinces that would incorporate videos with discipline-specific information about diverse options for staying independent at home. As the module is interactive, people can watch only the videos relevant to their needs, and watch them again or watch others if their needs change.

## Methods

### Study Design and Context

We used a 3-phase, multiprong, user-centered design [22] involving a needs assessment with qualitative interviews (phase I), module development (including script, videos, and textual information; phase II), and acceptability and usability testing (phase III). The study was approved by the CSSS Alphonse-Desjardins (Lévis) Ethics Committee and University of Alberta Health Research Ethics Board (Pro00055678).

Because health care in Canada is delivered under provincial and territorial rather than federal health insurance plans, we focused on 2 Canadian provinces: Quebec, a largely French-speaking province in eastern Canada with over 8 million inhabitants including 1.5 million seniors, and Alberta, an English-speaking province in western Canada with over 4 million inhabitants and almost 500,000 seniors. English and French versions of the module were developed simultaneously.

We were guided by a multidisciplinary steering committee of experts in SDM (MG, FL), primary care (FL), rehabilitation (AJ), architecture (NR), intensive care (PA), and a caregiver (LB), who met during each phase of the research and were responsible for data collection and analysis, determining the content of the module, and designing it. Video development, structure, and content were informed by the Interprofessional Shared Decision Making (IP-SDM) model [23,24], one of the few SDM models to acknowledge the contribution of multiple stakeholders, including multidisciplinary health teams and caregivers, in informing individuals’ health-related decisions [25-29]. It has already proven useful in multiple contexts, including in decisions about where frail seniors will reside [18,25,26,29-31]. We added architects and urban planners, who reflected on the importance of neighborly relations and familiarity with one’s surroundings in decisions about keeping seniors independent in their communities [18,26,29,32-37] (personal communication from Roy et al, 2017).

### Participants

We recruited convenience samples of potential end users of the module (seniors, caregivers, and health care professionals) from Quebec and Alberta. Participants were identified using snowballing [38], based on our steering committees’ social and professional networks.

*Seniors* were included if they were 65+ years and had struggled with how to remain independent at home. *Caregivers* (eg, son, daughter, or spouse) were included if they cared for a senior who had faced decisions about maintaining independence at home. Participants were excluded if they were cognitively impaired or not able or willing to sign informed consent. *Professionals* could be any professional with clinical experience in maintaining seniors at home, as well as built environment experts [39]. Teams caring for seniors could include caregiver representatives, home support workers, family doctors, home care nurses, nutritionists, social workers, pharmacists, physiotherapists, or occupational therapists.

### Phase I: Needs Assessment

#### Data Collection

Inspired by other needs assessments [13], we conducted single, semistructured interviews to assess information needs for decision making about housing options (from lay and professional perspectives) among seniors, caregivers, and health professionals (see Textbox 1 for the interview guide). Interviews were conducted by 2 trained female research assistants with expertise in health care research. No prior relationship was established with participants other than a call or email contact to set a date for the interview. Field notes were taken. According to participants’ preferences, interviews were conducted by phone or face-to-face in a place convenient for them. Interviews were conducted in French or English. Written informed consent was obtained. Participating seniors and caregivers received C$15 to cover expenses.

#### Data Analysis

Interviews were audio-recorded and transcribed verbatim. They were descriptively content coded by a researcher using a deductive approach (coding emerged from the data), and then checked by the research assistants who had conducted the interviews. We listed and then produced an overview of the most important information needs (factors most mentioned) for decision making about housing options, that is, options that could best support staying independent at home, barriers or facilitators, costs, and relevant sources of information. In collaboration with team members, these information needs were organized into 6 categories: cognitive autonomy, psychological or mental well-being, functional autonomy, social autonomy, financial autonomy, and people involved. The categories emerged from the data and were congruent with literature on stated reasons for institutionalization [40-47] and on the concept of *positive health* [48]. Most factors (codes) were mentioned by multiple respondents (saturation). No software was used, as we planned no analysis other than listing the needs (factors considered important for maintaining independent at home) to ensure that they were all present in the module.

### Phase II: Development of the Module

#### Script

We linked information needs to solutions (options) and labeled them according to the IP-SDM model (focusing on identifying the decision and people involved, definition of their role, and providing information about options, including benefits, risks, and consequences) to ensure that they were addressed in the videos [49]. Video scripts were drafted and finalized by our steering committee. Participants from phase I were asked to provide feedback.

#### Videos

Interactive videos presented the options that best responded to the decision-making needs identified by end users. In the videos, the 15 stakeholders relate personal anecdotes, but include balanced, evidence-based information [50]. Videos can facilitate thinking and problem solving using verbal, nonverbal, and visual communication techniques [51,52] and can contribute to (shared) decision making [50]. They have proven appropriate for less literate populations [53] and people with vision or hearing disabilities. As the module is interactive, people can watch only the videos relevant to their needs, and watch them again or watch others if their needs change. Concrete (local) options as per province are further explained in a separate section of the website with links to more information sources. Users are invited to update and comment on this information using a comments tool. A webmaster approves all posts before publication and updates information.

#### Web-Based Decision Support

Although some evidence supports the use of the Internet by seniors [54-56], there are also concerns [57,58]. However, seniors will be increasingly computer literate as time passes [54-56], and the speed at which new options become available makes offline information provision (eg, paper brochures) inefficient. This Web-based module is accessible on computers, tablets, or mobile phones on demand (any time or place). It can easily be updated and host different media. Housing decisions need constant reevaluation as seniors’ physical or mental health deteriorates [40], and Web-based decision support allows visitors to select the information relevant for their decisional stage, their personal or situational context, and their physical and mental functioning.

### Phase III: Usability Testing

#### Participants

Participants were seniors, caregivers, and professionals who participated in phase I, and new participants recruited by snowballing and networking of team members. Participants were invited by personal email or by phone, and with an advertisement on the Arthritis Society of Canada website (Joint Health Express). The survey was also available on the interactive website itself (but not mandatory for visitors). A completed questionnaire was considered informed consent. No incentives were offered.

#### Data Collection

Participants were sent a Web-based questionnaire with instructions to view the interactive website at their own pace and convenience, and then answer 14 questions about its acceptability and usability (Textbox 2) [15].

**Interview guide.**

Seniors/caregivers

Introduction

- What is your/your loved one’s year of birth?
- What is your/your loved one’s living situation?
- Do you/your loved one currently receive home care? (If so, which?)
- Which resources have you/your loved one added to your home to keep living there?
- Which community resources are available to you/your loved one and which do you/your loved one use?
- Can you tell me briefly what you think about your/your loved one’s situation and whether you have ever thought about moving or trying to stay as long as possible at home, and how?
- (For caregivers only) What is your relationship with the senior?
- Can you tell me about the extent to which you are involved in the care of your loved one, and decision making about social and medical decisions?

Main

- As you/your loved one have grown older, what are the important factors to keep mobile (independent) in your/your loved one’s home and your community? (ie, who/what helps you stay mobile/independent?)
- What do you think are the most important issues associated with staying independent at home (issues that should be addressed in our videos)?
- Do you know anything about your options, and the cost of each, in terms of staying at home or moving? Where did you/your loved one get information about these things?
- Did anybody ever bring up the question whether or not to stay at home or move with you/your loved one?

Professionals

Introduction

- What is your profession?
- Home or acute care?
- What is the percentage of older people in your clientele?
- How many years have you worked in this profession?
- Can you give me a short example of what the job is about (something you would tell a senior when they come to see you and do not know what you do)?

Main

- From your (professional) point of view, what factors or issues are important when considering mobility/independence in the home or community for older adults?
- What options are most often available for seniors for remaining mobile and independent at home, from your (professional) point of view?
- What do you think are the most important issues (issues that should be addressed in our videos)?
- Can you give an estimate of costs of the options that you mentioned?
- How do people get reimbursed for this?

**Usability test.**

- Please rate each section of the module by circling one of the following to show what you think about the clarity of the information: 4—Everything clear, 3—Most things clear, 2—Some things unclear, and 1—Many things unclear.
- The length of each separate video was: Too short/Just right/Too long.
- The total time needed to watch all the videos was: Too short/Just right/Too long.
- The amount of information was: Too little /Just right /Too much.
- I found the presentation: Slanted toward staying at home/Balanced/Slanted toward moving elsewhere.
- Do you think the module would be helpful for people making this decision? Yes/No
- Do you think the module would be acceptable to use with people making this decision? Yes/No
- Did the full module (including the videos) meet your expectations?
- Would you like to use (or keep using) the module?
- What did you like about the module?
- What did you dislike about the module? (concerns)
- At what point would it be useful for seniors to see this module? When they are still able to function at home without help/When they are beginning to lose autonomy /When they can no longer function on their own at home/Other, please specify.

All questions were posted on the same page, and answers could be reviewed and changed until submission of the questionnaire. One reminder was sent after 4 weeks.

#### Data Analysis

We performed a descriptive analysis of the data, and calculated mean, median, and range when relevant. Summary statistics were performed using Microsoft Excel.

## Results

### Phase I: Needs Assessment

#### Study Population

Between March 20 and September 28, 2015, we interviewed 29 stakeholders: 15 in Alberta and 14 in Quebec. Participants included 4 seniors, 3 caregivers, and 22 health care and other professionals (see Table 1). None of those contacted refused to participate. One interview was conducted simultaneously with both members of a senior couple. The mean interview time was 31.45 min (range 11.42-69.47).

#### Important Factors Influencing Decision Making About Staying Independent at Home

##### Cognitive Autonomy

Respondents mentioned the need for seniors to think about future housing options before there is cognitive decline or an emergency (eg, a fall). Seniors also should be encouraged to consider their changing needs over time to avoid having to frequently reevaluate them. This way, when the time comes to relocate, they may avoid long waiting lists for assisted living facilities and adjust more easily to the new environment. At the same time, seniors may be well aware of risks, and what to do about them should be in their hands.

##### Psychological or Mental Well-Being

Depression among seniors was frequently mentioned. It may be associated with isolation, as well as with the general effects of aging and loss of autonomy. Depression medication is associated with reduced mobility and risk of falls, whereas social or exercise programs (eg, walking or gardening) may improve both mental and physical health. It was mentioned that all who are involved in decision making should encourage seniors to participate in activities that they enjoy. Participants also mentioned the importance of seniors being happy and feeling safe where they are, but that they must accept that their needs are changing before they decide to make adjustments (eg, use a walker). Having a positive attitude toward the options for staying at home and being involved in the decisions about them were considered important decisional needs. Attitudes often change once people have tried the equipment and seen the benefits for themselves.

##### Functional or Physical Autonomy

Functional autonomy encompasses physical ability (muscle mass, strength, and balance), the environment, and prevention and support. If seniors are less able to perform certain tasks or activities, instead of doing the task for them, it is important to teach them a new way to perform the task themselves. Autonomy also depends on their home environment. Environmental barriers indoors (furniture, carpets, clutter, and distance to the toilet in case of incontinence) and outdoors (curbs and stairs) should be assessed in terms of safety. In suggesting equipment such as walkers or grab bars, it is important to discuss with seniors what is important for them and what limits them from being active. Specialized architects and occupational therapists can suggest how to redesign the living environment, such as moving the bedroom and toilet downstairs. Urban planners, who are more concerned with the external environment, pay attention to curbs and other walkability features of cities and public spaces. However, it should be recognized that whatever adaptations one makes to the home and environment, choosing to stay independent at home may one day no longer be realistic, safe, or affordable.

**Table 1:** Sociodemographic characteristics of participants in phase I (N=29).

Characteristics			Quebec	Alberta	Total
**Seniors**			2	2	4
	Age, range		87-88	82-84	82-88
	Sex (female), n (%)		1 (50)	1 (50)	2 (50)
	**Marital status, n**				
		Married	2	1	2
		Widowed	-	1	1
	**Living situation, n**				
		House or suburban or urban area	2	1	2
		Apartment style condo or urban	-	1	1
**Caregivers**			1	2	3
	Mean age, range		67 (N/A)^a^	69 (56-82)	68.3 (56-82)
	Sex (female), n (%)		1 (100)	2 (100)	3 (100)
	**Relation with senior, n**				
		Spouse	-	1	1
		Child	1	1	2
	**Living situation, n**				
		House with senior or urban	1	1	2
		Bungalow with senior or urban	-	1	1
**(Health) professionals**			11	11	22
	**Type of professional, n**				
		Dietitian	1	1	2
		Physiotherapist	2	2	4
		Occupational therapist	2	1	3
		Social worker	1	1	2
		Family physician	1	-	1
		Transition nurse in geriatrics	1	-	1
		Geriatrician	1	1	2
		Architect	1	1	2
		Human resources consultant (community activities)	1	-	1
		Pharmacist	-	1	1
		Recreational therapist	-	1	1
		Nurse or case manager in homecare	-	1	1
		Coordinator at community organization that helps the elderly	-	1	1
	Sex (female), n (%)		10 (91)	10 (91)	20 (91)
	Years of experience, range		15.2 (5-33)	16 (5-31)	15.6 (5-33)
	Percentage of elderly clients, range		82.3 (40-100)	77 (30-100)	79.5 (30-100)

^a^N/A: not applicable.

It is also important to assess people’s mode of transportation to access services in the community. People who drive their own cars are often more active, but at some point, this may no longer be safe. Many special seniors’ transport options exist such as buses for people with disabilities, taxis, as well as special seniors’ services that offer drivers to accompany seniors to their medical appointments.

##### Social Autonomy

When people age, their contemporaries start to die, leaving some people feeling isolated. Being socially active was mentioned several times. Although not everyone minds being alone, many benefit from having company and participating in activities with others. Social participation and having a social network are safer (somebody to call for help) and can be an information source about options. Many seniors are reluctant to ask for help, which means admitting to themselves and others that they are no longer autonomous. This reluctance can also make caring for them more difficult.

##### Financial Autonomy

To receive the help they need at home, seniors in many jurisdictions have to be willing and able to pay for services. Although some services are provided or reimbursed through government programs or tax credits, other services require direct payment from the senior. To be reimbursed, the senior must have the mental agility and patience to fill out difficult forms or have somebody to help him or her. With the Internet, it is increasingly possible to order and pay for things online and have them delivered (groceries, medication, or clothes) to the home, but many seniors are not able to do this, whether from lack of a computer, knowledge, or confidence.

##### People Involved

Seniors, caregivers, and health professionals need to understand each other’s limits and communicate about their difficulties. As informal caregivers are often key to keeping seniors independent at home, they should be kept informed of everything regarding the senior’s health care needs and options that help them stay at home. Without a single care coordinator, it is often the informal caregiver who manages the patient’s *file*. Their needs should be taken into account too, as the burden can become too much for them and threaten their own health. Caregivers can be informed of caregiver associations and helped to gain access to services such as respite care.

Table 2 provides the quotes from participants, and Table 3 provides an overview of the factors.

### Phase II: Development of the Module

#### The Script

On the basis of the factors identified in phase I, we developed scripts for 15 stakeholders in decisions about seniors’ housing options: a decision coach, a senior, 3 types of caregivers (son, daughter, or spouse), a caregiver representative, a home support worker, a family doctor, a home care nurse, a nutritionist, a social worker, a pharmacist, a physiotherapist, an architect, and an occupational therapist. The decision coach explained how a decision should be made and introduced the rest of the videos, and the architect added information about community, the built environment, and home adaptation. We kept the scripts as general as possible so that the information would not quickly become outdated and that it could be used in several contexts. Information that frequently changes, such as costs and resources, was presented on the resource page.

In total, 4 stakeholders commented on the script (caregiver, pharmacist, architect, and physiotherapist). Overall, they were positive about it. On the basis of their expert opinion, we made some editorial changes to the script and the list of resources, and a senior (female, 85 years old, living independently at home with her daughter) evaluated the final scripts. She was positive about them and the initiative as a whole, and she thought the information was complete and relevant.

#### The Final Module

The final product is an interactive website with video links and additional text-based resources for seniors, caregivers, and professionals, called *SupPortIng seNiors And Caregivers to stay mobile at Home* (SPINACH). It consists of 3 Web pages as discussed below.

##### Homepage

Visitors select English or French, and then choose whether to see the videos, consult the resources, or comment on or add to the resource page.

##### Video Page

A team of 15 members sitting around a table shows visitors the range of stakeholders involved in making decisions with seniors who wish to stay independent at home (Multimedia Appendix 1). A mouse scroll-over function presents a short description of what each team member will discuss in his or her 1- to 3.5-min video. After selecting a team member, a popup appears with the videos (Multimedia Appendix 2). The selected stakeholder talks about his or her decision-making experiences with staying at home or moving, provides information on decisions related to staying at home, and/or gives specific information on options or guidance in the decision-making process.

##### Resource Page

The resource page provides with background information on options for staying independent at home (eg, local resources, equipment, and links to informative websites). Visitors can add comments or additional information about local services. A webmaster evaluates them and controls publication.

**Table 2:** Quotes illustrating the main categories from phase I.

Theme		Quotes	
Cognitive autonomy		*And if people want to live with a degree of risk, that's fine from my stand point. As long as they understand that [the risk and consequences]. I mean we can't wrap people up, you know in lots and lots of bubble wrap to totally protect them.* [A13, Geriatrician]	
		*Yes and it’s, as I say, if you make the choice yourself it makes it easier for everybody. When you get to that point in life where you have to, and you don’t have a choice and you have to go where you are sent, I think a lot of people have trouble adjusting.* [A7 and A8, Seniors]	
**Psychological or mental well being**			
	Depression		*So what factors do you think are important to consider when trying to keep them mobile, I guess, in the community? [Interviewer]* *Finding things they like to do. Like if they love animals, like walking the dog. If they like, you know, the river valley, take them to the river valley. Finding things to engage them so that they want to do it, not so they have to do it… [Participant]* *You know, for us we say as long as it's something active, it can be anything. You know, we can go for a walk and they love doing crossword puzzles, maybe we stop at a park, do some crossword puzzles.* [A14, Coordinator in a home care company]
	Attitude		*[…] when you go to a walker, you are giving up your dignity. […] You are no longer totally independent. Here I am, I am handicapped now, I cannot just stride off into the sunset. I need help. It’s hard for people to understand the lack of enthusiasm for suddenly having to start using a cane and a walker.* [A5, Senior]
			*Many seniors don't want to look like seniors. They don't want to look old. You know, even though it might improve their ability to be mobile.* [A13, Geriatrician]
			*I would say the bigger challenge is when someone is adamant that they will lose independence because of the walking aid and once they’ve tried it you can often convince people that actually when they use the walker, you can actually walk further because you can sit down when you are tired, you do not have to look for a bench to sit on. […] but it can be difficult to persuade people that they need to give it a try because a lot of people just cannot get past the stigma of seeing themselves as someone who is using a walker.* [A2, Physiotherapist]
**Functional or physical autonomy**			
	Physical functioning		… *if you don’t use it you lose it! I get so tired of that phrase, but it’s so true. And there is the social aspect [of exercise programs] as well, which is maybe almost as important as the mobility factor.* [A5, Senior]
	Environment		*You want to age in your current location, you are attached to it, have memories but you have difficulties in moving around, using stairs, or you are anxious […]. My role is to see how we can modify this environment, do renovations, to make sure that you can be autonomous, that you feel safe and good in your home. It is possible that if there is nothing that can be done at the regulatory level, in terms of financial resources or support in your environment (family, neighbours) then the best option may be to move. But we know how hard it is to leave your house so we are here to help you.* [R5, Architect specialized in housing for the elderly]
	Transport		*Can she (the senior) go out, take the bus, walking, what are the distances to walk in the suburbs to do the grocery shopping and bring back bags with groceries? Is there adapted transportation? And the time you have to wait before it comes to your house? Is there a taxi? Maybe it is less expensive to take a taxi than to move to a residence with services.* [R5, Architect specialized in housing for the elderly]
Social autonomy		*Like let’s start doing this, you know, while you’re healthy, while you're well, because then, you know once they’ve got that social network then that just opens up so many more doors.* [A11, Occupational therapist in home care]	
		*It’s through social contacts really that you know about things. It’s important that you know people, and they tell you about these things.* [A5, Senior]	
		*A lot of it, you know like so many seniors, they say oh my kids are so busy, they can't do this, and they’ve got a very important job and all of that kind of stuff. But we need to actually kinda break it down, you know, the family members are the ones that...they certainly would do something if they knew what to do. Or if they knew that things would be a little better for their loved ones.* [A11, Occupational therapist in home care]	
People involved		*Some families are great and some are not but you need to be able to see that your supports are there and that they are functioning and healthy and that it is not taking its toll on one single person. But it often does.* [A6, Social worker]	

**Table 3:** Overview of the categories and factors mentioned in the interviews (phase I).

Category, factor (code), and examples (subcodes)			Solutions	Barriers to implementation
**Cognitive autonomy**				
	**Good judgment regarding risks**			
		Estimate risks, being self-critical	Avoid risks	Attachment to belongings
	**Good judgment and decision making**			
		Incompetent	Power of attorney, personal directive	Needs to be put in place before cognitive decline
		Competent: making decisions yourself makes adapting easier	Incorporate everyone’s values and preferences	
	**Memory**			
		Remember to eat, take meds, and turn off oven	Caregiver, calendar, box for pills, and microwave instead of oven	Isolation, confusion
	Thinking ahead			People do not know what their needs will be in 5-10 years
**Psychological or mental well-being**				
	**Happiness**			
		Happy at home	Stay at home	Functional incapacity, isolation
	**Feeling safe, fears**			
		Stays in because is afraid to walk outside	Motivation, support people	Attitude—not willing to use aids or ask for help
	**Depression**			
		Due to isolation, general effects of aging, loss of autonomy	Medication, exercise programs, and caregiver	Medication can affect mobility; lack of awareness about benefits of participation for mental and physical health
**Functional autonomy**				
	**Managing the basic needs**			
		Medication	Community and social care services, check with pharmacist	Medication interactions with comorbidity
		Hygiene	Grab bars in bathroom, care services offered by public health care system	Adaptability of homes, attitude of seniors
		Food preparation and access (quantity and quality)	Meal services, vitamin D supplements and calcium	Attitude—willingness or ability to cook Cost of meal services or ready cooked meals
	**The body**			
		Good muscle mass, cardiorespiratory	Exercise program, services at home	Lack of motivation, accessible programs, awareness, education, or confidence; fear of falling; focus on disability, pain versus on ability
	**Getting around (internal and external)**			
		Design of the environment	Remove architectural barriers (internal: furniture, carpets; external: sidewalks, stairs)	Attitude: do not want change, want to design own house Costs of home renovations
		Transport	Bus or taxi for seniors or caregiver transport	Access to information, costs
		Mobility	Walkers, canes	Attitude: “those are for old people,” giving up dignity and independence
		Pay attention to environment	Height of curb, height and number of stairs, and rugs	Awareness of need to change habits (people already use the walls and counters for balance or support), muscles that have not been used for a long time, current habits not safe
	**Prevention**			
		Of falls, eating problems	Programs, equipment, awareness; learning new things that they can still do themselves instead of taking it out of their hands	
**Social autonomy**				
	**No isolation, well-being**			
		Hobbies, activities	Day centers, friends	People have habits and do not like to change. Try to find intrinsic motivation, but decision is up to them
	**Have people around you**			
		Friends, family, neighbors	Social worker or caregiver	Not everybody wants to meet other people and do things together
	**Ask for help (formal and informal)**			
		Emergency system, lifeline	Bracelet, call someone	Attitude: children are too busy
**Financial autonomy**				
	**Ability to pay and manage finances**			
		Pension, reimbursement, subsidies	Financial support, tax benefits, and health insurance	Income: not enough to pay for services Attitude: unwillingness to pay, inability to complete forms for reimbursement, mental capacity, online payments versus cash
**People involved in caring for senior**				
	**Good collaboration**			
		Caregiver and health care professionals understand each other’s limits, communicate		Do not get on with social worker
	**Caregiver needs**			
		Decrease the burden	Respite care Access to care when caregiver is not around	Access to information, capacity to advocate, family member differences regarding how they view the senior

### Phase III: Acceptability of the Module

A total of 21 people completed the acceptability survey: 8 seniors, 7 caregivers, and 6 professionals. Respondents were mostly female (15/21). Mean age of seniors was 76 years (range 66-91), whereas the mean age of caregivers was 69 years (range 36-70; Table 4). Most seniors and caregivers had higher education (college or university; 12/15). Caregivers were mostly adult children of a senior parent. Seniors had either no caregivers (n=3), an adult child (n=2), a partner (n=2), or other (n=1). Professionals were a nurse, an urbanist, an architect, a social worker, and a community care worker. In addition, 3 other professionals gave feedback by email to the researcher.

#### Comprehensibility

With a mean overall rating of 3.6 out of 4, participants thought the videos were very clear (Table 4), although one senior suggested that content should better reflect differences across Canada.

#### Length and Amount of Information

Most participants were positive about the length of each video (17/21; 81%) and the amount of information (17/21; 81%), but fewer people liked the length of the videos altogether (13/21; 62%). Some thought the module was too long (n=7) or had too much information (n=2), whereas others wanted more information (n=2). Others indicated that the module could be better tailored to specific characteristics of the senior (n=4).

**Table 4:** Acceptability test results for seniors, caregivers, and professionals (N=21).

Acceptability test question			Seniors (n=8)	Caregivers (n=7)	Professionals (n=6)	Total group (N=21)
**Comprehensibility of videos (mean rating out of 4)**^a,b^						
	Video: decision coach		3.7	3.8	3.5	3.7
	Video: senior 1		3.9	4	3.7	3.8
	Video: caregiver 1		3.4	3.8	3.7	3.6
	Video: caregiver 2		3.5	3.8	3.7	3.6
	Video: caregiver 3		3.9	3.8	3.7	3.7
	Video: caregiver representative		3.3	3.7	3.7	3.5
	Video: home support worker		3.6	3.8	3.7	3.7
	Video: family physician		4	3.8	3.3	3.7
	Video: nurse in homecare		3.7	3.8	3.3	3.6
	Video: dietitian		3.8	4	3.7	3.8
	Video: social worker		3.8	3.7	3.2	3.5
	Video: pharmacist		3.7	4	3.7	3.7
	Video: physiotherapist		3.9	4	3.7	3.8
	Video: architect		3.6	4	2.8	3.4
	Video: occupational therapist		3.6	4	3.2	3.6
**Length of single videos, n (%)**						
	Too short		-	-	1 (17)	1 (5)
	Just right		7 (78)	5 (83)	5 (83)	17 (81)
	Too long		2 (22)	1^b^ (17)	-	3 (14)
**Time needed to watch all videos, n (%)**						
	Too short		-	-	1 (17)	1 (5)
	Just right		4 (44)	5 (83)	4 (67)	13 (62)
	Too long		5 (56)	1^2^ (17)	1(17)	7 (33)
**Amount of information, n (%)**						
	Not enough		1 (11)	-	1(17)	2 (9)
	Just right		7 (78)	5 (83)	5 (83)	17 (81)
	Too much		1 (11)	1^b^ (17)	-	2 (9)
	Presentation was balanced, yes		6 (67)	5 (83)	6 (100)	17 (81)
	Helpful? yes		6 (67)	5 (83)	5 (83)	16 (77)
	Acceptable to use for people in this situation? yes		8 (89)	5 (83)	5 (83)	18 (86)
	Did module meet your expectations? yes		6 (67)	5 (83)	5 (83)	16 (77)
	Would you like to use/keep using the module? yes		3 (34)	3 (50)	6 (100)	12 (58)
	**At what point would it be useful for seniors to see this module?, n (%)**					
		When they start losing their autonomy	3 (34)	2 (33)	3 (50)	8 (38)
		When they are still able to function at home without help	6 (66)	4 (67)	3 (50)	13 (62)

^a^4—Everything clear; 3—most things clear; 2—some things unclear; 1—many things unclear.

^b^One caregiver did not watch any videos, and rated all as *Not applicable*.

#### Helpfulness

Most respondents thought the module was helpful (16/21; 77%). In total, 4 people did not agree because of navigation difficulties (1 senior), inadequate detail, 1 professional), lack of specificity or tailoring (1 senior), or design (1 caregiver). Of the participants, 1 senior was not prepared to accept her need to make any adaptations, so the information was of no interest to her.

#### Acceptability

Most respondents thought the module was acceptable (18/21; 86%), but 3 people did not because of a lack of information (1 professional), too idealistic presentation of options (1 caregiver), and navigation difficulties (1 caregiver). All professionals were eager to start using the module with their clients when finalized (6/6; 100%), as were most caregivers (3/7; 43%). Seniors were less willing to continue using the module (5/8; 66%) because of navigation difficulties or because it needed changes (n=2), or their preference for information face-to-face or in a brochure (n=2). A senior said she would return to the module when it becomes more relevant to her situation.

#### What People Liked About the Module

Participants liked the interprofessional character of the module, the diversity of information, the concrete and practical examples, and the information about available services. They also liked the positive tone (focus on what can be done instead of what cannot).

#### Concerns About the Module

The concern most often mentioned by seniors was its overall length. A senior and a caregiver mentioned that it did not portray the reality (eg, long wait times or services that are not available). A caregiver was negative about the whole module, saying he did not like watching videos. Some professionals wanted more details on the role of health professionals (eg, health evaluations by nurses). Others mentioned the challenge of using the module with people with dementia.

## Discussion

### Principal Findings

Using a 3-phase, user-centered design, we developed and tested the acceptability of an interactive Web-based module for seniors, their caregivers, and health professionals with videos presenting information about options for staying independent at home. A needs assessment (phase I) uncovered numerous decisional needs, including the need to start thinking about this decision early on, safety issues inside and outside the home, and the importance of social supports and psychological or mental well-being. In the production phase (phase II), we developed scripts that addressed these decisional needs, which were positively evaluated by end users and used to create 15 bilingual videos. These were integrated into an interactive Web-based module. In usability testing (phase III), this was found to be clear, comprehensible, and providing enough information, but users found that it took too long to watch all of the videos and that seniors might need assistance navigating the module. In total, 3 concluding reflections were derived from these findings.

First, research has shown that good levels of knowledge about services and support, as well as good housing, are associated with the likelihood of continuing to live in the community [59]. In accordance with our needs assessment, the content of the module focused primarily on the first steps in SDM models: identifying the decision and people involved and providing information about options, including benefits, risks, and consequences [13,27]. Decisions made in the absence of knowledge about available options are less likely to be accepted (phase I) and may result in feelings of uncertainty and conflict in the caregiver or conflict between the caregiver and the senior [9]. For a full SDM process, an interactive (face-to-face) discussion with all those involved in decision making is required to weigh all relevant information in light of personal and professional opinions and reach agreement about the best option [31,60]. As the Internet has been found to be one of the most commonly used and trusted information sources for health information among the elderly [56], this Web-based module can prepare people for this discussion. As decisional needs and Internet use vary with personal and sociodemographic characteristics [15,61], our videos present a variety of senior and caregiver profiles, and the viewer can select which ones to watch. However, acceptability test results led us to conclude that much shorter videos tailored to different characteristics could provide people with information even more closely matched to their needs, increase understanding, and decrease viewing time [20]. Future plans with this module are to tailor the information and videos to characteristics and situations, such as early symptoms of dementia, physical problems, and presence and type of caregiver. In line with concerns of participants who were uncertain about whether (other) seniors would be able to go through the module alone [58,62], we suggest that seniors go through the module with a caregiver. This would also facilitate personal dialogue between the senior and the caregiver and improve the process of SDM.

Second, staying active and being socially engaged were mentioned to be linked with staying healthy in both the needs assessment and the acceptability test. The concept of positive health refers to the capacity to live autonomously with physical, emotional, and social challenges [48]. This relates to the principles of self-management and *use it or lose it* mentioned by our participants (phase I), reflected in the videos (phase II), and positively evaluated in the acceptability test (phase III) [63]. This module helps seniors to self-manage and stay independent by helping them think about what they can and cannot do by themselves. Although seniors in our study showed less concern for social support, many professionals in our study mentioned its importance for well-being and autonomy, and other research supports this [64]. Social support may be a crucial and overlooked element of the inside and outside built environment and of community care options for seniors. The Internet may be another way to access social support [55].

Third, the module emphasizes the importance of involving seniors *and* caregivers *and* professionals in decision making, implying that they all need to know and understand the best available evidence regarding the risks and benefits of all options for staying independent at home [17,60,65]. This interdisciplinary aspect of our module, which was specifically appreciated by participants and is considered key in IP-SDM [16-18], was possible because of the interdisciplinary and user-centered design used throughout the development of the module. Although future research should assess what types of end users should be involved in updating the module and to what extent, we will continue to involve end users in updating the module by inviting them to add regional information or propose adaptations using the comment function. This function will facilitate ongoing and sustainable patient and professional engagement in our process [66,67]. The Web-based and end user–adaptable nature of the module will also facilitate implementing and scaling up the intervention [68].

### Limitations

Although people were initially enthusiastic about participating in several developmental rounds, the decreasing response per round indicated that more was needed to successfully create ownership and that participants were losing interest because of the lengthy process.

Data collection and identification of local resources took place in Quebec and Alberta only. Thus, although the videos are bilingual, generic, and in principle applicable to any context, local options are currently available only for Quebec and Alberta. Future studies could adapt the module to other Canadian contexts. Finally, the modest number of participants in this project was adequate for a user-centered development process, but future phases of this project should use different study designs (eg, pre-post or randomized trial designs) and larger samples of participants to achieve generalizable results, for example, with regard to the effectiveness of the module or its implementation.

### Conclusions

We adopted a user-centered design to develop a Web-based decision support module for seniors, caregivers, and health professionals that incorporates discipline-specific information about options for staying independent at home. The module was deemed acceptable and potentially helpful.

Seniors are often anxious and fearful when the first signs of loss of autonomy appear, and confused about what they can do about it. These are first steps toward providing them with the information they and their caregivers need to make decisions about how to stay independent at home. We plan to adapt the module to better target the most important user (the senior), and to continue its development and evaluation, as well as develop implementation strategies. In the meantime, the module is available on the Web as the information on the module is already of use for many seniors.

## Appendix

Multimedia Appendix 1 Screenshot of stakeholders whose videos can be chosen using mouse scroll-over.

Multimedia Appendix 2 Screenshot of a stakeholder (architect).

## Abbreviations

IP-SDM: Interprofessional Shared Decision Making
SDM: shared decision making
