# Supplementary figures and images for: Deciding How to Stay Independent at Home in Later Years: Development and Acceptability Testing of an Informative Web-Based Module
Source: JMIR Hum Factors. 2017 Dec 14;4(4):e32. doi: 10.2196/humanfactors.8387 (PMC5746619; doi:10.2196/humanfactors.8387)

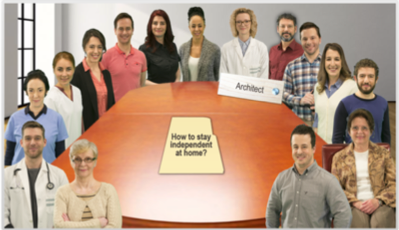

Supplement: Multimedia Appendix 1 [file humanfactors_v4i4e32_app1.png]

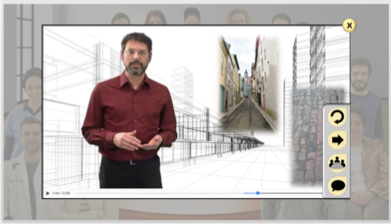

Supplement: Multimedia Appendix 2 [file humanfactors_v4i4e32_app2.png]
